# Prevalence of unintended pregnancy and associated factors among pregnant mothers in Jimma town, southwest Ethiopia: a cross sectional study

**DOI:** 10.1186/s40834-019-0090-4

**Published:** 2019-07-08

**Authors:** Girma Alemayehu Beyene

**Affiliations:** Department of Public Health, College of Health Science and Medicine, Wolkite University, Wolkite, Ethiopia

**Keywords:** Unintended, Mistimed, Unwanted, Unplanned, Pregnancy

## Abstract

**Background:**

Unintended pregnancy is a pregnancy that occurred when no children were desired or earlier than desired. One in three births in Ethiopia were unintended, which is major contributor to maternal morbidity and mortality. The objective of this study was to assess prevalence of unintended pregnancy and associated factors among pregnant women in Jimma.

**Methods:**

Community based cross-sectional study was conducted from May to June, 2016. Estimated sample size of 356 was allocated proportionally to the randomly selected kebeles based on number of pregnant women in their kebeles and simple random sampling technique was used in selecting participants. Data were collected by structured questionnaires with face to face interview, analyzed by SPSS and multivariate logistic regression was fitted to identify factors independently associated with unintended pregnancy.

**Results:**

The prevalence of unintended pregnancy among the study participants was 36.5% (95% CI: 31–42%), of which 97 (27.2%) were unwanted and 33 (9.3%) were mistimed. The most common reasons for failure to avoid unintended pregnancy were because they believed they were not fertile 54 (41.5%) and 26 (20%) became pregnant despite the use of contraceptive methods. Age of the mother over 35 years, lower educational status of the partner, higher family size, smaller number of children desired and less knowledge of modern contraceptive methods were independent predictors of unintended pregnancy.

**Conclusion:**

The prevalence of unintended pregnancy was high in the study area and programs aimed at reducing unintended pregnancy should target older couple. In addition, improving educational status of the partner and knowledge of modern contraceptive methods is demanded.

## Background

Unintended pregnancy is a pregnancy that is either unwanted or mistimed. Unwanted pregnancy is pregnancy that occurred when no children were desired and mistimed pregnancy is the one that occurred earlier than desired [1]. In spite of availability and widespread information how to use modern contraceptive methods, unintended pregnancy is one of the most common reproductive health problems faced by women of child bearing age.

Globally, there were an estimated 99.1 million unintended pregnancies per year, of which 21.6 million were in Africa and Eastern Africa alone encompasses 8.85 million. Worldwide, an estimated 44% of pregnancies and 23% of births were unintended and 56% of all unintended pregnancies ended in abortion. Likewise, 39% of all pregnancies in Africa and 46% of pregnancies in Eastern Africa were unintended. Similarly, 27% of births in Africa and 37% of births in Eastern Africa were unintended [2].

Despite decline of total fertility rate to 4.6 children per woman in 2016, there is high prevalence of unintended childbirth in Ethiopia. Nearly one in three (32%) of births in Ethiopia were unintended; and about two-thirds of them were mistimed [3, 4]. Prevalence of unintended pregnancy among pregnant women in Oromia region ranges from 37.3% in Bale zone to 27.1% in Gelemso Hospital [5–8]. More than third of pregnant mothers in Southern Ethiopia reported that their most recent pregnancy was unintended [9–11].

Unintended pregnancy mainly results from not using contraception, or inconsistent or incorrect use of effective contraceptive methods [1]. Younger age at marriage, lower educational and occupational status of the mother, history of previous unintended pregnancy, low awareness and use of contraceptive methods, husband refusal to use family planning, less number of children desired, low awareness of the concept of unintended pregnancy is preventable, high parity were factors significantly associated with unintended pregnancy [7, 10, 12, 13].

Couples ability to have the number of children they want when they want them is central to the quality of their lives and has vital significance for the future of the family and the world. Planning the number and timing of pregnancy may give the time to be ready physically, economically and mentally for healthy pregnancy and childbirth, recover easily after giving birth. Women with unintended pregnancies are less likely to seek prenatal care during the first trimester, more likely to use substances like alcohol and tobacco during pregnancy, have low birth-weight infants or abortion. In countries where abortion is illegal and unsafe, unintended pregnancy is a major contributor to maternal morbidity and mortality [14–16].

Limited studies have described the magnitude of unintended pregnancy and associated factors among pregnant mothers; even those few studies were conducted in health facilities among mothers attending antenatal or post-natal care which by itself affected by intentional status of pregnancy. Hence, this study tried to fill the aforementioned gap by conducting the study in community setting. Therefore, the aim of this study was to determine the prevalence of unintended pregnancy and associated factors among pregnant mothers residing in Jimma town, southwest Ethiopia. The finding of this study aims to guide health professionals, reproductive health program planners and policy makers as well as other concerned bodies to understand tackle unintended pregnancy targeting factors having significant association and reduce the risk of maternal and infant morbidity and mortality.

## Methods

### Study area and period

The study was conducted in Jimma town of Oromia regional state which is located at 346 km to South West of the national capital, Addis Ababa. According to the data obtained from Jimma town vital statistics office the total population of the town is 189,733 of which 96,764 are females and the rest are males. The town consists 19 kebeles (administrative units in Ethiopia) of which 13 are urban and 6 of them are rural kebeles. There are two hospitals, four health centers, 34 drug store, 113 clinics including private and non-governmental organizations and 15 health posts in the town. The study was conducted from May to June, 2016.

### Study design and population

Community based cross sectional study was conducted among pregnant women in Jimma town.

### Sample size

The sample size for the study was calculated using Epi Info™ version 7 StatCalc function of Sample Size calculation for population survey at 95% confidence interval (CI), 5% marginal of error, considering 36.5% of pregnant women reported unintended pregnancy from related study in Ganji woreda of west Wollega zone [7] which gave the largest sample size and adding 10% non-response rate, a total of 356 study participants were estimated for this study.

### Sampling technique

Six kebeles were selected randomly by lottery method. Total sample size was allocated proportionally to the selected kebeles based on number of pregnant women in their kebeles. Households with pregnant women were listed out from family folder of health extension workers (HEW) and the study participants were selected using simple random sampling technique specifically Excel generated random numbers.

### Data collection techniques and procedures

Data relevant for the study was collected by using structured questionnaires adapted from related studies and incorporating additional potential variables with face to face interview of the respondents by three trained health extension workers. The questionnaire was developed in English and translated in to local language Afaan Oromo to facilitate communication. Pregnancy intention were measured using the standard approach, which probes women to recall their feelings at the time they became pregnant [1].

### Data quality assurance

The quality of data collection was monitored by giving clear instruction to data collectors how to approach respondents and fill the questionnaire. Close supervision by Degree holder was made and filled formats and questionnaires were checked on daily basis for completeness and consistency. At the end of each day of data collection the completed questionnaire paper were checked for its consistency.

### Data processing and analysis

The collected data were cleaned, coded and entered into Epi Info™ 7 statistical software package and exported to SPSS version 22 for statistical analysis. Frequency distribution for selected variables was performed. Bivariate logistic regression analyses were conducted to nominate candidate variables for multivariable analysis and those with *p* ≤ 0.25 were included into the initial multiple logistic regression models, using backward fitting. Variables persisted to be associated with the outcome at *p* ≤ 0.05 were used and displayed in the final model. Adjusted Odds ratio (AOR) with its 95% CI was considered to judge for precision and decide whether independent association between outcome and independent variables exist. Finally, the results of the study were presented using texts, tables and figures based on nature of the data obtained.

### Operational definitions

**Unintended pregnancy:** pregnancy that occurred when no children were desired or that occurred earlier than desired [1].

**Unwanted pregnancy:** pregnancy that has occurred to the women who not wants to become pregnant neither at the time of conception nor in the future [16].

**Mistimed pregnancy:** pregnancy that occurred when a woman has a desire to be pregnant and have a child or children sometime in the future, but not now [7].

**Knowledge of contraceptive methods:** Ten knowledge questions about modern contraceptive methods were used and those who answered eight or more questions were categorized as more knowledgeable & those who answered less than eight were grouped as less knowledgeable [12].

**Partner:** A person with whom the woman had intimate sexual relationship and became pregnant with the index pregnancy.

## Results

### Sociodemographic characteristics

A total of 356 pregnant women responded to the questionnaire which makes the response rate of 100%. Most of the study participants, 167 (46.9%) of them were in age group of 26–35 years followed by those aged 36 years and more 108 (30.3%). The mean (Standard deviation (SD)) age of the study subjects was 30.89 (± 6.55) years. More than three fourth, 277 (77.8%) of the respondents were married and more than three fifth, 220 (61.8%) of them were followers of orthodox religion. Regarding educational status, 109 (30.6%) of the study participants attained primary education and 92 (25.8%) of them completed secondary education. More than half, 186 (52.2%) of the respondents were housewives. Concerning educational status and occupation of the partner 104 (29.2%) of them attained secondary education and 134 (37.6%) of them were self-employed (Table 1).

**Table 1:** Socio-demographic characteristics of study participants in Jimma, Ethiopia, 2016

Variables	Frequency	Percent
Age (Mean = 30.89 ± 6.55)		
≤ 25	81	22.8
26–35	167	46.9
≥ 36	108	30.3
Family size (Mean = 3.38 ± 2.17)		
0–3	189	53.1
4–6	136	38.2
> 6	31	8.7
Marital status		
Married	277	77.8
Single	8	2.2
Widowed	47	13.2
Divorced	24	6.7
Religion		
Orthodox	220	61.8
Muslim	85	23.9
Catholic	6	1.7
Protestant	45	12.6
Educational status of the respondent		
Can’t read and write	31	8.7
Read and write	70	19.7
Primary	109	30.6
Secondary	92	25.8
College & above	54	15.2
Occupation of the respondent		
Housewife	186	52.2
Student	32	9
Self-employed	86	24.2
Employee	52	14.6
Educational status of the partner		
Can’t read and write	35	9.8
Read and write	63	17.7
Primary	96	27
Secondary	104	29.2
College and above	58	16.3
Occupation of the partner		
Farmer	87	24.4
Student	4	1.1
Employee	117	32.9
Self-employed	148	41.6

### Reproductive history

The mean (SD) age the study subjects married was 25.03 (± 12.24) years. More than third, 127 (35.7%) of the participants were married at age of 20 or less. More than three fifth, 219 (61.5%) of mothers desired to have 4–6 number of children and only quarter 91 (25.6%) of them desired having more than six children. Among the study participants 127 (35.7%) of them had previously experienced unintended pregnancy and 91 (25.6%) of them had history of abortion (Table 2).

**Table 2:** Reproductive history of study participants in Jimma, Ethiopia, 2016

Variables	Frequency	Percent
Age at marriage (Mean = 25.03 ± 12.24)		
≤ 20	127	35.7
21–24	110	30.9
> 25	119	33.4
Number of pregnancies (Mean = 4.92 ± 2.52)		
< 4	117	32.9
4–6	146	41
> 6	93	26.1
Number of children desired (Mean = 5.41 ± 1.47)		
< 4	46	12.9
4–6	219	61.5
> 6	91	25.6
Previous unintended pregnancy		
Yes	127	35.7
No	229	64.3
Think unintended pregnancy is preventable?		
Yes	222	62.4
No	134	37.6
Antenatal care visit		
Yes	182	51.1
No	174	48.9
History of abortion		
Yes	91	25.6
No	265	74.4
Knowledge of contraceptive methods		
Less knowledgeable	160	44.9
More knowledgeable	196	55.1

### Prevalence of unintended pregnancy

**Fig. 1:**
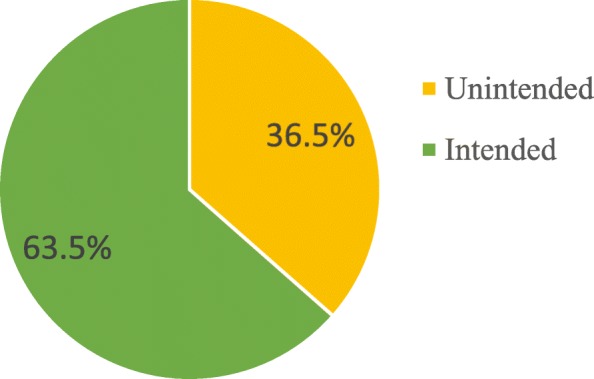
Pregnancy intention among pregnant women in Jimma town, Southwest Ethiopia, 2016

Among the pregnant mothers included in the study 97 (27.2%) of them conceived when no more child was desired (unwanted pregnancy) and 33 (9.3%) of them became pregnant earlier than they desired to be (mistimed pregnancy). This makes the prevalence of unintended pregnancy among the study participants to be 36.5% (95% CI: 31–42%). The remaining were conceived either at the right time or later than desired, hence the pregnancy was intended (Table 3 & Fig. 1).

**Table 3:** Magnitude of unintended pregnancy among study participants in Jimma, Ethiopia, 2016

Timing of current pregnancy	Frequency	Percent
Unwanted	97	27.2
Mistimed	33	9.3
Right time	167	46.9
Later than desired	59	16.6

### Perceived reasons for unintended pregnancy

**Fig. 2:**
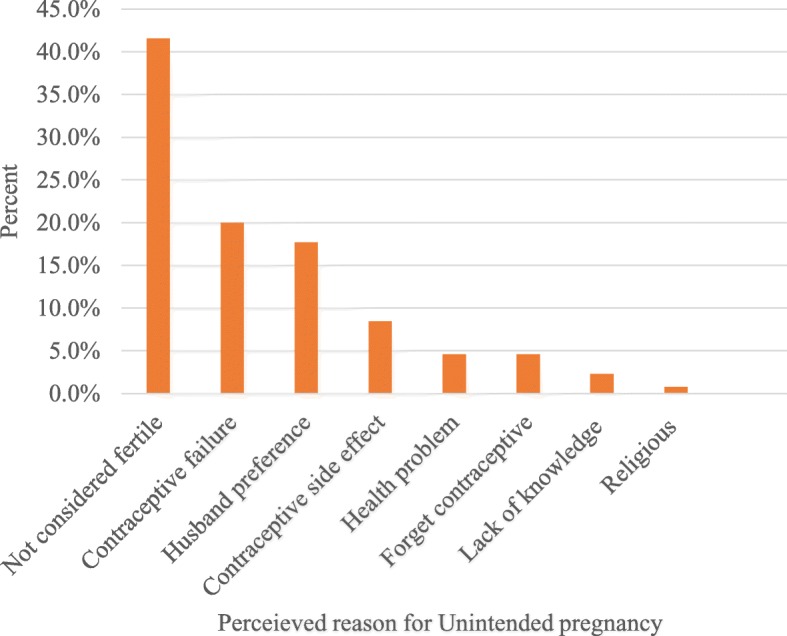
Perceived reasons for unintended pregnancy among pregnant women in Jimma town, Southwest Ethiopia, 2016

Among those who reported to have unintended pregnancy, the most common reason incriminated for failure to avoid unintended pregnancy were because they believed that they were not fertile 54 (41.5%) and one in five 26 (20%) of the mothers with unintended pregnancy became pregnant despite the use of contraceptive method due to contraceptive method failure. Twenty-three (17.7%) of mothers with unintended pregnancy became pregnant with-out their will because their husband prefers to have more children (Fig. 2).

### Factors associated with unintended pregnancy

From bivariate analysis age and educational status of the mother, educational status of the partner, family size, number of pregnancies, desired number of children, previous unintended pregnancy, history of abortion and knowledge of contraceptive methods were selected as a candidate variable for multivariate analysis so as to appreciate their independent effect. However, in multivariate analysis using multiple logistic regression age of the mother, educational status of the partner, family size, desired number of children and knowledge of modern contraceptive methods were found to be independently and significantly associated with unintended pregnancy.

After controlling for other independent factors maternal age was found to be significantly associated with unintended pregnancy. Mothers aged 36 years or more were nearly five times more likely to experience unintended pregnancy compared to mothers aged 25 years or less (AOR: 4.97; 95% CI: 2.53, 9.77).

Mothers whose partner can’t read and write were more than 14 times more likely to have unintended pregnancy when compared to those whose partner attained college and above (AOR: 14.59; 95% CI: 4.60, 46.31). Mothers with partner who can read and write (AOR: 3.62; 95% CI: 1.47, 8.93) and who attained primary education (AOR: 3.36; 95% CI: 1.42, 7.97) were more than three times more likely to experience unintended pregnancy compared to mothers whose partner attained college level or above.

Independent of other predictor variables, the higher the family size, the higher likely is the unintended pregnancy. Family having 4–6 members were four times more likely to have unintended pregnancy compared to those with three or less family members (AOR: 4.19; 95% CI: 2.01, 8.76). Family with more than six members were more than seven times more likely to have unintended pregnancy compared to family with three or less members (AOR: 7.63; 95% CI: 2.37, 24.52).

Those who desired to have less than four children were 2.89 times more likely to report unintended pregnancy compared to those who desired to have six or more number of children (AOR: 2.89; 95% CI: 1.25, 6.73). Those who desired to have 4–6 children were 2.11 times more likely to have unintended pregnancy compared to those who desired to have six or more number of children (AOR: 2.11; 95% CI: 1.34, 3.92).

Controlling for potential predictor factors, mothers who are less knowledgeable about contraceptive methods were nearly three times more likely to have unintended pregnancy compared to their counterparts (AOR: 2.92; 95% CI: 1.87, 4.56).

However, educational status of the mother, number of pregnancies, previous unintended pregnancy and history of abortion were not significantly associated with unintended pregnancy (Table 4).

**Table 4:** Bivariate and Multivariate analysis of factors associated with unintended pregnancy among pregnant mothers in Jimma, 2016

Characteristics	Categories	Unintended	Intended	COR (95% CI)	AOR (95% CI)	*P*-value
Age of the mother	≤25	22	59	1	1	
	26–35	34	133	0.68(0.37,1.27)	0.61(0.32,1.16)	0.13
	≥36	74	34	5.84(3.09,11.03)	**4.97(2.53,9.77)** ^**^	0.00
Educational status of the mother	Can’t read & write	18	13	5.41(2.05,14.33)	0.94 (0.10,8.42)	0.95
	Read and write	27	43	2.46(1.08,5.56)	0.54(0.07,4.23)	0.56
	Primary	43	66	2.55(1.18,5.48)	2.01(0.22,18.63)	0.54
	Secondary	31	61	1.98(0.90,4.38)	0.94(0.12,7.54)	0.96
	College & above	11	43	1	1	
Educational status of the partner	Can’t read & write	28	7	17.09(5.94,49.18)	**14.59(4.60,46.31)** ^**^	0.00
	Read and write	29	34	3.65(1.60,8.29)	**3.62(1.47,8.93)** ^**^	0.00
	Primary	33	63	2.24(1.03,4.88)	**3.36(1.42,7.97)** ^**^	0.00
	Secondary	29	75	1.65(0.75,3.62)	2.06(0.87,4.85)	0.10
	College & above	11	47	1	1	
Family size	0–3	37	152	1	1	
	4–6	71	65	4.49 (2.74,7.34)	**4.19(2.01,8.76)** ^**^	0.00
	> 6	22	9	10.04(4.27,23.61)	**7.63(2.37,24.52)** ^**^	0.00
Number of pregnancies	< 4	25	92	1	1	
	4–6	47	99	1.75(0.99,3.06)	0.68(0.31,1.47)	0.33
	> 6	58	35	6.09(3.32,11.22)	1.47(0.56,3.83)	0.44
Desired number of children	< 4	20	26	2.15(1.02,4.53)	**2.89(1.25,6.73)** ^*^	0.01
	4–6	86	133	1.81(1.05,3.09)	**2.11(1.34,3.92)** ^*^	0.02
	> 6	24	67	1	1	
Previous unintended pregnancy	No	65	164	1	1	
	Yes	65	62	2.65(1.68,4.15)	0.86(0.47,1.58)	0.63
Abortion history	Yes	40	51	1.53(0.94,2.48)	1.42(0.86,2.35)	0.17
	No	90	175	1	1	
Knowledge of contraceptive methods	More knowledgeable	50	146	1	1	
	Less knowledgeable	80	80	2.92(1.87,4.56)	**2.92(1.87,4.56)** ^**^	0.00

^**^: significant at *p* value < 0.00

^*^: significant at *p* value < 0.05

## Discussion

The prevalence of unintended pregnancy in this study was 36.5%: (95% CI: 31–42%), which is consistent with similar study conducted in Bale zone, Ganji, Duguna Fango, Debre-Markos, Hossana and Harar [5, 7, 8, 10, 11, 17]. But, higher than the study conducted in Arba-Minch [12], indicating higher rate of unintended pregnancy despite increased reproductive health services.

The most common reasons mentioned by women for failure to avoid unintended pregnancy are not considering oneself as fertile, 54 (41.5%) and contraceptive method failure 26 (20%). A similar finding was reported from a study in Gelemso, East Ethiopia [6]. Which indicated sustained gaps in creating awareness of reproductive aged women concerning appropriate utilization of modern contraceptives methods. Health education related to fertility has to be provided to reproductive age women, so that they understand the time they would be fertile.

Women who are less knowledgeable about modern contraceptive methods were more likely to have unintended pregnancy, which is consistent with the study done in Arba-Minch [12]. This might be explained by the fact that women with less knowledge about modern contraceptive methods were less likely to know the available, accessible and safe contraceptive methods and other reproductive health services. Therefore, less likely to utilize the method correctly and and hence more likely to have unintended pregnancy. As a result, it’s important to improve reproductive age women knowledge about modern contraceptive methods in order to tackle unintended pregnancy.

Unintended pregnancy was found to be higher among women older than 35 years. Which is inconsistent with study conducted in Gelemso hospital, Bale zone, Hossana and Brazil [5, 6, 10, 18] but consistent with the study done in Kersa and Ganji Woreda of West Wollega that showed higher unintended pregnancy among older women [7, 19]. This might be due to the fact that older women will not consider themselves as fertile, less likely to be educated and hence less knowledgeable about modern contraceptive methods and also more likely to have partner with lower educational status. Hence, programs aiming to reduce unintended pregnancy should consider older women as potential target.

Women with partner of lower educational status is more likely to report unintended pregnancy possible explanation for this might be partner with no formal education or lower educational status disagree with mother’s preference to use modern contraceptive methods and/ or less likely to encourage their wives to use family planning methods. Attitudes and behaviors of male partners may influence women’s intentions, sexual behavior, contraceptive use and parenting [1].

The finding of the study depicted the higher the family size, the higher likely is the unintended pregnancy, this is in line with the study conducted in Damote Gale, Southern Ethiopia and Debre-Markos Town, North West Ethiopia [9, 17]. The rational for this might be families with a greater number of children already reached their desired number of children and hence, any more pregnancy is likely to be unintended.

The lower number of children desired, the higher the risk of unintended pregnancy, which is consistent with the study done in Hossana town, Southern Ethiopia [10]. This might be due to the fact that the lower the number of children the mother desired, higher order pregnancies are more likely to be unintended.

## Conclusion and recommendation

The prevalence of unintended pregnancy was high in the study area being major reproductive health problems with all its adverse health outcomes to the mothers, children, families and societies at large. Age of the mother over 35 years, lower educational status of the partner, family size of more than four children, a smaller number of children desired and less knowledge of modern contraceptive methods were independent predictors of unintended pregnancy.

Teenagers and young adults were considered to be at higher risk of unintended pregnancy. As a result, unintended pregnancy was linked with teenage pregnancy. However, our finding indicated older women are at higher risk of unintended pregnancy and hence programs aimed at reducing unintended pregnancy should target the elders.

In addition to educating and empowering women, attention has to be given for educating male partner as most decisions in the household, including the timing of pregnancy and number of children desired, are strongly influenced by the male partner. Moreover, improving knowledge regarding the use of modern contraceptive methods is demanded.

Most women became pregnant because they did not consider themselves as fertile, so fertility education has to be provided to the older women so that they clearly understand the time in which they would be fertile. It is recommended that policy makers, health professionals, and health authorities should give due attention to the improvement in the provision of effective Information Education and Communication (IEC) and counseling and quality of care. Sexual and reproductive health education with more focus on fertility and contraceptive methods should be given for the couples, with more emphasis for the older as they are the less likely to achieve formal education.

## Data Availability

The datasets used and/or analyzed during the current study are available from the corresponding author on reasonable request.

## Abbreviations

AOR: Adjusted odds ratio
CI: Confidence interval
HEW: Health extension workers
IEC: Information Education & Communication
NGO: Non-governmental organization
SD: Standard Deviation
SPSS: Statistical Package for Social Science
